# Analysis of a new phage, KZag1, infecting biofilm of *Klebsiella pneumoniae*: genome sequence and characterization

**DOI:** 10.1186/s12866-024-03355-9

**Published:** 2024-06-14

**Authors:** Ebtsam Saqr, Mahmoud W. Sadik, Gamal El-Didamony, Ahmed Askora

**Affiliations:** 1 Department of Microbiology and Botany, Faculty of Science, Zagazig University, Zagazig, 44519 Egypt; 2 Department of Environmental Biotechnology, College of Biotechnology, Misr University for Science and Technology, Giza, Egypt; 3 Department of Microbiology, Faculty of Agriculture, Cairo University, Giza, 12613 Egypt

**Keywords:** Bacteriophage KZag1, *K. pneumoniae*, Biofilm reduction, Genomic analysis, Phage Therapy, And Antibiotic resistance

## Abstract

**Background:**

This study investigates the effectiveness of the bacteriophage KZag1 against drug-resistant *Klebsiella pneumoniae*, aiming to assess its potential as a therapeutic agent. The novelty lies in the characterization of KZag1, a Myovirus with specific efficacy against multidrug-resistant *K. pneumoniae* strains. This highlights the significance of exploring alternative strategies, particularly phage therapy, in addressing biofilm-associated infections.

**Methods:**

KZag1, characterized by a typical Myovirus structure with a 75 ± 5 nm diameter icosahedral head and a 15 ± 5 nm short tail, was evaluated in experimental trials against 15 strains of *K. pneumoniae*. The infection cycle duration was determined to be 50 min, resulting in an estimated burst size of approximately 83 plaque-forming units per colony-forming unit (PFU/CFU). Stability assessments were conducted within a pH range of 4 to 12 and temperatures ranging from 45°C to 60°C. Biofilm biomass reduction was observed, particularly at a multiplicity of infection (MOI) of 10.

**Results:**

KZag1 demonstrated infection efficacy against 12 out of 15 tested *K. pneumoniae* strains. The phage exhibited stability across a broad pH range and at elevated temperatures. Notably, treatment with KZag1 significantly reduced *K. pneumoniae* biofilm biomass, emphasizing its potential in combating biofilm formation. Genomic analysis revealed a complete genome of 157,089 base pairs with a GC content of 46.38%, encompassing 203 open reading frames (ORFs) and a cysteine-specific tRNA sequence. Comparison with phage GP4 highlighted similarities, with KZag1 having a longer genome by approximately 4829 base pairs and a higher GC content by approximately 0.93%. Phylogenetic analysis classified KZag1 within the *Myoviridae* family.

**Conclusion:**

The efficacy of KZag1 against *K. pneumoniae* biofilm suggests its potential as a therapeutic candidate, especially for drug-resistant infections. Further clinical research is warranted to explore its synergy with other treatments, elucidate genomic traits, compare with *Myoviridae* phages, and understand its host interactions. These findings underscore the promising role of KZag1 in addressing drug-resistant bacterial infections.

## Introduction

*Klebsiella pneumoniae*, a Gram-negative, non-motile, encapsulated bacterium, is a member of the *Klebsiella* genus within the Enterobacteriaceae family. It is responsible for various infections including pneumonia, urinary tract infections, and nosocomial infections. Respiratory tract infections caused by *K. pneumoniae* are widespread and severe, with a high mortality rate reaching 50% [1]. The situation is further complicated by the prevalence of antibiotic-resistant strains, with approximately 80% of the *K. pneumoniae* isolates being resistant to antibiotics, making treatment challenging [2]. The rise of multidrug-resistant strains, particularly those resistant to carbapenems, has become a significant issue in both hospital and community settings due to the extensive use of antibiotics and efficient transmission [3]. Additionally, *K. pneumoniae* is notable for its ability to form biofilms, which contributes to its significance as a major pathogen in healthcare-associated infections [4]. Biofilm formation in *K. pneumoniae* infections is a crucial virulence factor, facilitating its persistence in clinical settings. Biofilms provide protection against the host immune response and antibiotics, promoting the acquisition of resistance traits and the development of multidrug resistance (MDR) phenotypes. The dense extracellular matrix of biofilms physically obstructs antibiotic penetration, while metabolic changes render bacteria less susceptible to antibiotics targeting actively dividing cells. Additionally, biofilm-associated gene expression upregulates efflux pumps and stress response mechanisms, further enhancing antibiotic resistance [4–6]. The emergence and rapid spread of multidrug-resistant strains of *K. pneumoniae* have posed a significant threat to public health, highlighting the urgent need for innovative approaches to combat these infections [7, 8]. Bacteriophages, or phages, represent a promising alternative to conventional antibiotics due to their specific targeting of bacterial pathogens [1–9]. These viruses have gained attention for their ability to specifically target and kill bacterial pathogens, while leaving beneficial bacteria and human cells unharmed [2, 7–10]. Phages are naturally occurring entities that infect and replicate within bacteria. They possess a high degree of specificity, with different phages targeting specific strains or species of bacteria [3–10]. This specificity is attributed to the recognition and binding of phage tail structures to specific receptors on the surface of bacterial cells.One of the key advantages of phages over traditional antibiotics is their ability to evolve alongside bacteria. As bacteria develop resistance mechanisms against antibiotics, phages can adapt and evolve to overcome these resistance mechanisms [4–9]. This dynamic nature of phages allows them to maintain their effectiveness against evolving bacterial pathogens [11] Moreover, phages have a unique mode of action compared to antibiotics. Instead of directly killing bacteria, phages replicate within the bacterial host, leading to the lysis and destruction of the bacterial cell. This lytic activity not only kills the targeted bacteria but also helps prevent the development of bacterial resistance [12]. Phages offer several other advantages as well. They have a broad range of host specificity, allowing them to target a wide variety of bacterial pathogens [13]. Additionally, phages can penetrate biofilms, which are protective structures formed by bacteria that make them highly resistant to antibiotics [14, 15]. By effectively targeting biofilms, phages provide a potential solution to chronic and persistent infections [5]. Another benefit of phages is their relative safety. They have been extensively studied and used in certain regions for decades, particularly in Eastern Europe, as a therapeutic option for bacterial infections. Phages are generally well-tolerated by the human body and have minimal impact on the normal microbiota [10–12]. Phage therapy, the use of phages to treat bacterial infections, has shown promising results in both in vitro and in vivo studies. It has demonstrated efficacy against multidrug-resistant bacteria, including those that are resistant to conventional antibiotics. However, further research is needed to fully understand the potential of phage therapy and optimize its use in clinical settings. The aim of this study is to characterize the genome sequence of phage KZag1 and investigate its potential as a therapeutic agent against biofilms of *K. pneumoniae*.

## Material and methods

### Bacterial strains and growth conditions

Clinical specimens suspected to contain *Klebsiella* isolates were collected from the sputum of patients at Zagazig University Hospital, Sharqia, Egypt, following the acquisition of informed consent. The collected samples were promptly transported to the laboratory for further processing. To ensure the safety of laboratory personnel and prevent cross-contamination, the samples were handled in accordance with standard biosafety protocols. Laboratory personnel wore appropriate personal protective equipment (PPE), including gloves and laboratory coats, during sample processing.

Serial dilutions of the collected samples were performed to achieve a suitable bacterial load for isolation. Using a sterile loop or spreader, the diluted samples were streaked onto selective agar plates specifically designed for the isolation of *Klebsiella*, such as MacConkey agar or blood agar (Oxoid, United Kingdom) [16]. These plates were then incubated at the optimal temperature for *Klebsiella* growth, typically 35–37°C, for a period of 24–48 h. Following incubation, the agar plates were examined for the presence of bacterial colonies. Colonies displaying characteristic *Klebsiella* morphology, such as a mucoid appearance, lactose fermentation, and non-hemolytic on blood agar plates, was selected. Using a sterile loop, these selected colonies were streaked onto fresh agar plates to obtain pure cultures, ensuring the isolation of individual *Klebsiella* strains [17]. The isolated colonies were then incubated under appropriate conditions to facilitate further growth and identification. To confirm the identity of the isolated *Klebsiella* strains, additional tests were conducted. Specifically, DNA extraction was conducted using the QIAamp DNA Mini Kit supplied from QIAGEN (USA) with Catalogue no. 51304. Subsequently, 16S rRNA sequencing was performed using the ready reaction Bigdye Terminator V3.1 cycle sequencing kit (Perkin-Elmer/Applied Biosystems, Foster City, CA), with Cat. No. 4336817, provided by Sigma Company, located in Giza, Egypt, for the accurate identification of the *Klebsiella* strains [18]. The GenBank accession number link provided: https://www.ncbi.nlm.nih.gov/nucleotide/OP942216.1.

### Antibiotic susceptibility test

The antibiotic susceptibility test was conducted on the clinical isolates to determine the susceptibility pattern of bacteria. The antibiotic sensitivity test was conducted using the Kirby-Bauer method (also called the disc diffusion test) on Muller-Hinton agar [12]. Sterilized Müller-Hinton agar (Oxoid, USA) was poured into Petri dishes in 10 mL aliquots. Once the agar solidified, 100 µL aliquots of broth cultures, inoculated with 10^8^ CFU mL^−1^ of the tested bacterial strains, were evenly spread over the surface of the agar plates. Antibiotic discs were carefully placed on the plate surfaces. Fifteen bacterial isolates were tested against the following antibiotics: Imipenem (IPM) (10 μg), Polymyxin B (PB) (300 μg), Amikacin (AK) (30 μg), Erythromycin (E) (15 μg), Trimethoprim/sulphamethoxazole (SXT) (25 μg), Cefoxitin (FOX) (30μg), Cefepime (FEP) (30μg), Ceftriaxone (CRO) (30μg), Ceftazidime (CAZ) (30μg), Gentamicin (CN) (10μg), Ciprofloxacin (CIP) (5μg), Erythromycin (E) (15μg), Chloramphenicol (C) (30mcg), Piperacillin (PRL) (100 μg), and Tetracycline (TE) (30 μg). The plates were then incubated upside down at 37°C for 16–18 h. The diameter of the inhibition zone around each antibiotic disc was measured in millimeters (mm). The interpretation of the inhibition zone diameter was classified as sensitive (S), intermediate (I), or resistant (R) based on the interpretative criteria recommended by CLSI [19] for antimicrobial susceptibility testing. Additionally, the multiple antibiotic resistances (MAR) index was calculated to determine the level of antibiotic resistance. The MAR index is the ratio between the number of antibiotics to which the bacteria are resistant and the total number of antibiotics used, as described by Sayah et al. [20].

### Enrichment and Isolation of *K. pneumoniae* bacteriophage

Bacteriophages were isolated from different sewage water samples obtained from Sharkia Governorate, Egypt, using the enrichment technique [21]**.** Initially, 100 mL of sewage was filtered through a 0.45μm-filter membrane and combined with an equal volume of nutrient broth in 500 mL Erlenmeyer flasks. Subsequently, 5 mL of fresh *K. pneumoniae* culture (2.0 × 10^8^ CFU mL^−1^) was added to each sewage sample. The flasks were incubated on a shaker (120 rpm) at 37 ºC for 24 h. After incubation, the mixture was centrifuged at 10,000 rpm for 20 min, and the resulting supernatant was filtered through a 0.45μm-filter membrane to detect the presence of phages using spot test and plaque assay methods with *K. pneumoniae* as the host. Phage titration was performed by tenfold serial dilution of the samples in saline solution and spotting them on lawns of *K. pneumoniae*, with the phage titer expressed in plaque forming units (PFU) per mL.

### Purification of *K. pneumoniae* bacteriophage

Phages were propagated and purified from various single-plaque isolates following Kim et al. [21]. The isolated phages underwent five successive single-plaque isolations until homogenous plaques were obtained. In each isolation, a single plaque was picked and incubated with 1 mL of nutrient broth containing an overnight culture of *K. pneumoniae* at 37 ºC with agitation. After incubation, the phage-host mixture was centrifuged at 10,000 rpm for 10 min, and the supernatants were filtered through a 0.45μm Millipore filter to eliminate any bacterial contamination. The purified phages were stored at 4ºC for further characterization.

### *Electron* microscopy analysis of isolated bacteriophage

The morphology and structure of the isolated bacteriophage were analyzed using transmission electron microscopy (TEM), according to the method described by Abdel-Haliem and Askora [22].The phage suspension was prepared, containing a concentration of 10^8^ (PFU mL^−1^). A small volume of the phage suspension was applied onto Carbon-coated formvar films on 200 mesh copper grids. Excess liquid was carefully removed from the grid using filter paper. A few drops of the Sodium phosphotungstate solution were added onto the grid, covering the phage particles. Excess Sodium phosphotungstate solution was gently removed from the grid using filter paper. Images of the phage particles were captured using a Hitachi H600A electron microscope at the Faculty of Agriculture, Mansoura University, Egypt.

### The one-step growth

The one-step growth curve of the phage was determined following a standard protocol [16]**.** Briefly, a culture of the host bacterium *K. pneumoniae* was grown to the logarithmic phase of growth. The bacterial culture was then infected with the phage at a multiplicity of infection (MOI) of 0.01, which means that for every bacterium, only one phage was added. After allowing the phage to adsorb to the bacterial cells for a specific duration 5 min, the mixture was diluted and plated onto agar plates to determine the number of viable phages (PFU) at time zero. This represented the initial phage count. Subsequently, samples were taken at regular intervals every 5 min for certain duration 2 h. Each sample was immediately diluted and plated onto agar plates to quantify the number of infective phages at each time point. This allowed for the construction of the growth curve. To calculate the burst size, the number of new phages released from each infected bacterium was determined by comparing the phage count at the peak of the growth curve with the initial phage count. The burst size represents the average number of phages released per infected bacterium. The experiment was performed in triplicate to ensure the reliability of the results, and the average values along with standard deviations were reported.

### Host range

The host range of Kzag1 phage was determined experimentally using a collection of bacterial strains, including *K. pneumoniae* and related species, as potential hosts. Each bacterial strain was cultured to the logarithmic growth phase, and 100 µL of each culture was spread on separate agar plates. After drying, small drops of the phage suspension were added to the agar surface to allow phage attachment to the bacterial cells. The plates were then incubated at the optimal growth temperature for the bacterial strains at 37°C. After an appropriate incubation period 18–24 h, the plates were inspected for the presence of plaques, indicating successful phage infection and subsequent bacterial cell lysis. The absence of plaques indicated that the phage could not infect the specific bacterial strain.

### The influence of temperature and pH on the stability of the phage

The influence of temperature and pH on the stability of the phage was examined. Thermal stability tests were performed following the protocol described by Mahmoud et al. [23]. Phage samples with a known titer (10^6^–10^8^ PFU mL^−1^) were exposed to different temperatures ranging from 30°C to 100°C for 10-min intervals in a water bath incubator. The infectivity of the phages was determined immediately after incubation using the double-layer agar plate method. Additionally, pH stability tests were conducted by inoculating a known phage suspension (10^6^–10^8^ PFU mL^−1^) into LB liquid medium obtained from Thermo Fisher Scientific, United States with pH values ranging from 3.0 to 12.0, followed by overnight incubation at 4°C. The viability of the bacteriophages was assessed using the overlay method as described by Adams [21]**.**

### The impact of the Kzag1 bacteriophage on the proliferation of *K. pneumoniae*

The impact of Kzag1 phage on the growth of *K. pneumoniae* was investigated. A culture of *K. pneumoniae* was diluted to a final density of 2.0 × 10^8^ CFU mL^−1^and placed in a nutrient broth, then incubated at 37°C for 24 h. The phage suspension was also diluted to achieve a MOI of 0.1. Subsequently, 100 µL of various phage concentrations was added to the bacterial suspension [24], and the mixture was incubated under sterile conditions. The survival of *K. pneumoniae* was assessed at intervals of 5, 10, 15, 20, 25, 30, and 35 min using the plaque assay method.

### The impact of individual Zag1 phage on biofilm formation

To assess the impact of Zag1 phage on biofilm formation by *Klebsiella*, the following experimental procedures were conducted according to Jamal et al. [25]. A biofilm was developed by introducing 200 μL of bacterial culture (10^8^ CFU mL^−1^) into each well of a 96-well flat-bottomed polystyrene microtiter plate, followed by incubation at 37°C for 24 h with gentle agitation at 120 rpm. After the biofilm formation period, any excess fluid was discarded, and the wells were washed twice with 0.9% NaCl to eliminate unattached planktonic cells. The plate was then allowed to dry at 37°C for one hour. The isolated phages were diluted in 0.9% NaCl and added to the respective wells containing their host bacteria. Different concentrations of the Zag1phage was used, including MOI = 0.1, MOI = 1, and MOI = 5. The control wells were set up using uninoculated normal saline, and they remained untreated throughout the experiment. Specifically, the uninoculated normal saline serves as the negative control, while the well inoculated with bacterial culture serves as the positive control. The plates were incubated at 37°C with constant shaking at 120 rpm for an additional day after the establishment of the biofilm. Excess fluid was removed from each well, and the biofilms were washed as previously described. The wells were left to dry for one hour at 37°C. To determine the total biomass of the biofilm, staining with 1% crystal violet was performed for 20 min. The plates were then washed with distilled water and air-dried. Next, 200 μl of 0.9% NaCl solution was added to each well, and the absorbance was measured at OD570 using an ELISA plate reader (Biotek Synergy HT Microplate Reader, USA. Triplicate measurements were taken for both control and test samples.

### Phage DNA sequencing and subsequent bioinformatics analysis

The phage DNA sequencing and subsequent bioinformatics analysis were carried out as follows: Initially, phage DNA was extracted using the phenol–chloroform method, following the protocol by Sambrook and Russell [26]. The purified phage DNA was then subjected to sequencing using the Illumina Miseq platform (Illumina, San Diego, CA, United States). The obtained sequencing data was assembled using SPAdeS v.3.13.0 [27] by Sanigen Inc., South Korea. To identify ORFs, a combination of Glimmer3 [28], GeneMarkS [29], and the RAST annotation server [30] was utilized. The annotated data were organized using Artemis [31]. Furthermore, the tRNA sequence within the phage genome was analyzed using the tRNAscan-SE program. Predictions for the functions of the phage proteins were made using NCBI BLASTp and the InterProscan program [32]**.** The annotated genome sequence of the KZag1 phage was deposited in the NCBI GenBank database under accession number OR502445. Phylogenetic analysis of the Zag1 phage was conducted by querying the Blast database and reconstructing a phylogenetic tree. Genomic sequences of relevant phages were retrieved from the GenBank database (https://www.ncbi.nlm.nih.gov/genbank/) [32]. These sequences underwent alignment using Clustal Omega to ensure precise alignment, considering sequence homology and structural similarities [33]. Subsequently, the aligned sequences were used to construct phylogenetic tree employing the robust maximum likelihood (ML) methodology [34]. Statistical analyses were conducted to evaluate the significance of the inferred phylogenetic relationships.

### Statistical analysis

Each experiment was conducted in triplicate, and the average of the triplicate determinations was taken to represent the results. Statistical analyses were carried out using SPSS software package version 11.5 and Microsoft Excel 2010. The data were subjected to analysis of variance, and significant differences (*p* > 0.05) between means were determined according to Pallant [35]**.**

## Results

### Antibiotic susceptibility of *K. pneumoniae* K9 (OP942216)

Among the 95 bacterial isolates obtained from sputum samples, 15 isolates (15.7%) displayed characteristic *Klebsiella* morphology, including mucoid appearance, lactose fermentation, and non-hemolytic on blood agar plates. These isolates were further selected for antibiotic susceptibility testing. The susceptibility of the isolated *K. pneumoniae* strains to various antibiotics was evaluated using the disc diffusion method. The results, shown in (Table 1), indicated that the *K. pneumoniae* strains exhibited resistance to more than two of the tested antibiotics. Notably, the strains showed resistance to multiple antibiotics, including gentamicin, sulfamethoxazole, tetracycline, amoxicillin/clavulanate, nalidixic acid, and cefotaxime. Among the tested isolates, *K. pneumoniae* K9, isolated from a sputum sample, displayed the highest Multiple Antibiotic Resistance (MAR) index, with a value of 1.0, indicating significant resistance to multiple antibiotics. Molecular identification of this isolate (K9) was performed by extracting genomic DNA, amplifying rRNA using PCR, and sequencing the amplicon. The obtained sequence was deposited in the GenBank under accession number OP942216.

**Table 1:** Antibiotic Susceptibility using disc diffusion method for Klebsiella pneumoniae

Antibiotic Class	Antibiotic	Bacterial Isolates														
		**K9**	**5D**	**5A**	**5B**	**5C**	**K8**	**6A**	**6B**	**6C**	**6D**	**K2**	**K11**	**K12**	**K17**	**K20**
**Cephalosporines 2nd generation**	**Cefoxitin (FOX 30µg)**	R	R	R	R	R	R	R	R	R	R	R	R	R	R	R
**Cephalosporines 3rd generation**	**Cefepime (FEP 30µg) Ceftriaxone (CRO 30µg) Ceftazidime ( CAZ 30µg)**	R R R	R R R	R R R	R R R	R R R	R R R	R R R	R R R	R R R	R R R	R R R	R R R	R R R	R R R	R R R
**Aminoglycosides**	**Gentamicin (CN 10µg) Amikacin (AK 30µg)**	R R	R R	S R	S R	R R	S R	R R	I R	R R	R R	I S	S S	S I	S S	R I
**Carbapenem**	**Imipenem (IPM 10µg)**	R	R	R	R	R	R	S	R	I	R	S	S	S	S	S
**Flouroquinolones**	**Ciprofloxacin (CIP5µg)**	R	R	R	R	R	R	R	R	R	R	S	R	R	S	R
**Macrolides**	**Erythromycin (E 15µg)**	R	R	R	R	R	R	R	R	R	R	R	R	R	R	R
**Chloramphenicols**	**Chloramphenicol** **(C30mcg)**	R	R	R	R	S	R	R	S	R	R	S	R	S	S	S
**Penicillins**	**Piperacillin (PRL 100 µg)**	R	R	R	R	R	R	R	R	R	R	R	R	R	R	R
**Tetracyclines**	**Tetracycline (TE 30mcg)**	R	R	R	R	R	R	S	S	S	S	S	S	S	S	R
**Sulfonamide**	**Trimethoprime/ Sulphamethoxazole** **(SXT25 µg)**	R	R	R	R	R	R	R	R	R	R	R	I	S	S	R
**Lipopeptides**	**Polymixin B (PB 300 IU)**	R	R	S	S	S	S	S	S	S	R	R	R	R	R	R
**Resistance percentage for each isolate (%)**		**100**	**100**	**85.7**	**85.7**	**85.7**	**85.7**	**78.5**	**71.4**	**78.5**	**92**	**57**	**64**	**57**	**50**	**78.5**
**MAR Index**		**1**	**1**	**0.8**	**0.8**	**0.8**	**0.8**	**0.7**	**0.7**	**0.7**	**0.9**	**0.5**	**0.6**	**0.5**	**0.5**	**0.7**

### Bacteriophage isolation and morphological characterization (TEM)

**Fig. 1:**
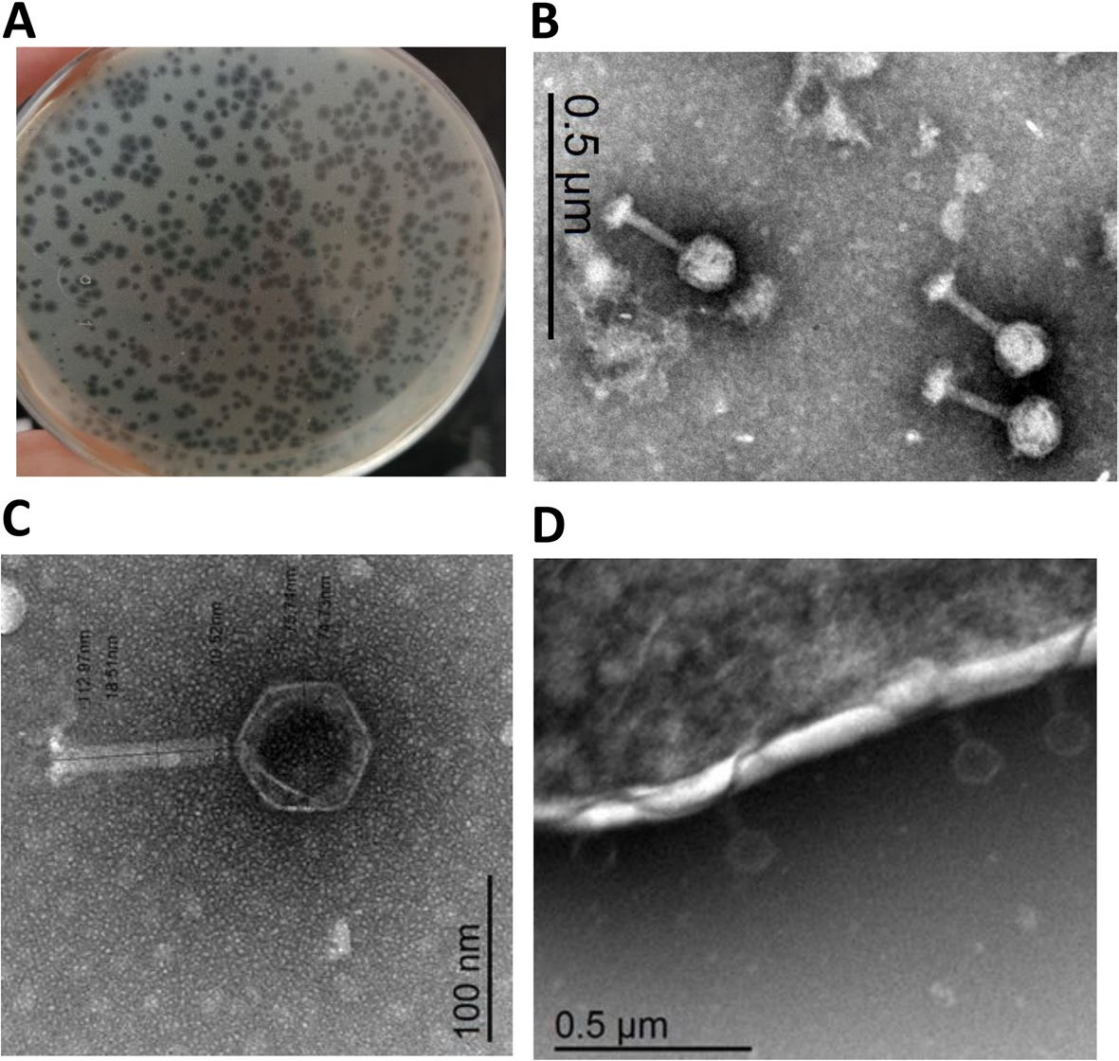
Morphological Characteristics of *K. pneumoniae* Phage KZag1 (**A**) Phage Zag1 Plaque Morphology NA double-layer agar plate showing the plaque morphology of phage Zag1. Clear zones indicate areas where phage KZag1 has lysed the host *K. pneumoniae* cells.** B** Transmission Electron Microscopy (TEM) Image of Phage Zag1 shows three individual phage Zag1 particles, with a scale bar of 0.5 µm. **C** Transmission Electron Microscopy (TEM) Image of Phage Zag1 High-resolution TEM image revealing the detailed structure of phage Zag1. Scale represents 100 nm. **D** Phage Zag1 Adsorption on *K. pneumoniae* Cell Visual depiction of phage Zag1 attached to the surface of a *K. pneumoniae* bacterial cell

Bacteriophages specific to *K. pneumoniae* K9 were isolated from an enrichment culture containing *K. pneumoniae*. Sewage water samples were collected from two large sewage plants in Sharkia Governorate, following the procedures outlined in the materials and methods section. The spot and plaque assay methods were utilized to detect the presence of phages. Among the observed plaques, a single plaque was selected for further analysis. This plaque was designated as Zag1, and subsequent purification and characterization steps were conducted. Transmission electron microscopy (TEM) was employed to examine the morphology of the isolated phage, KZag1. KZag1 exhibits a typical Myovirus structure; featuring a 75 ± 5 nm diameter icosahedral head and a short tail measuring 15 ± 5 nm.The results demonstrated that KZag1 belongs to the *Myoviridae* family. (Fig. 1).

### Host range of *K. pneumoniae* KZag1 phage

The infectivity of Zag1 phage was assessed against different strains of *K. pneumoniae* and other bacterial strains (Table 2). KZag1 phage displayed robust lytic activity specifically targeting *K. pneumoniae* (Table 2)**.** However, the remaining tested bacterial strains showed resistance to infection by the isolated phage, underscoring the highly specific nature of KZag1 towards *K. pneumoniae*.

**Table 2:** Host Range of *K. pneumoniae* KZag1 phage

Bacterial host	Strain	Spot test
*Klebsiellapneumoniae*	K9 (main host)	** + ve**
*K. pneumoniae*	K2	+ ve
*K. pneumoniae*	K8	-ve
*K. pneumoniae*	K11	-ve
*K. pneumoniae*	K12	+ ve
*K. pneumoniae*	K17	+ ve
*K. pneumoniae*	K20	-ve
*K. pneumoniae*	5A	+ ve
*K. pneumoniae*	5B	+ ve
*K. pneumoniae*	5C	+ ve
*K. pneumoniae*	5D	+ ve
*K. pneumoniae*	6A	+ ve
*K. pneumoniae*	6B	+ ve
*K. pneumoniae*	6C	+ ve
*K. pneumoniae*	6D	+ ve
*Staphylococcus aureus*	saEg01LC596095	-ve
*Escherichia coli*	M30LC649234.1	-ve
*Escherichia coli*	ATCC25922	-ve
*Salmonella typhi*	ATCC14028	-ve
*Pseudomonas aeruginosa*	ATCC9027	-ve

### One-step growth curve of KZag1 phage

**Fig. 2:**
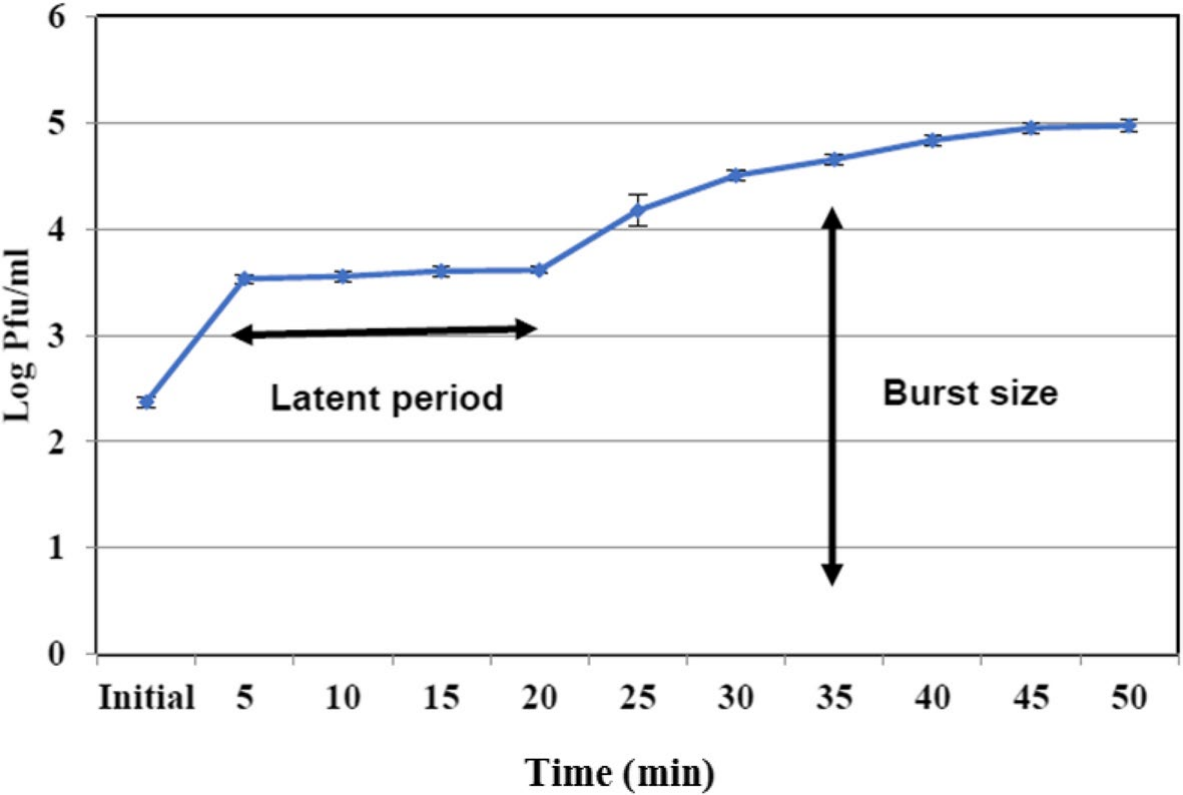
Single-step growth curve for *K. pneumoniae* KZag1 phage. The plaque forming units (PFUs) per infected cell in cultures of *K. pneumoniae* K9 at different time post infection are shown. Samples were taken at intervals every 10 min

The one-step growth curve analysis of KZag1 phage revealed a characteristic pattern of phage infection and replication. The latent period, which represents the time between phage adsorption and the initiation of replication, was determined to be 20 min. This was followed by a rise period lasting 35 min, during which the phage population experienced exponential growth (Fig. 2). The entire cycle of infection, from adsorption to the release of new phage particles, was completed in approximately 50 min. The burst size, indicating the number of phage particles released per infected host cell, was calculated to be 83 for KZag1 phage. These findings provide valuable information about the kinetics and efficiency of phage replication and release within the host bacterial population.

### Effect of temperature and pH on KZag1phage stability

**Fig. 3:**
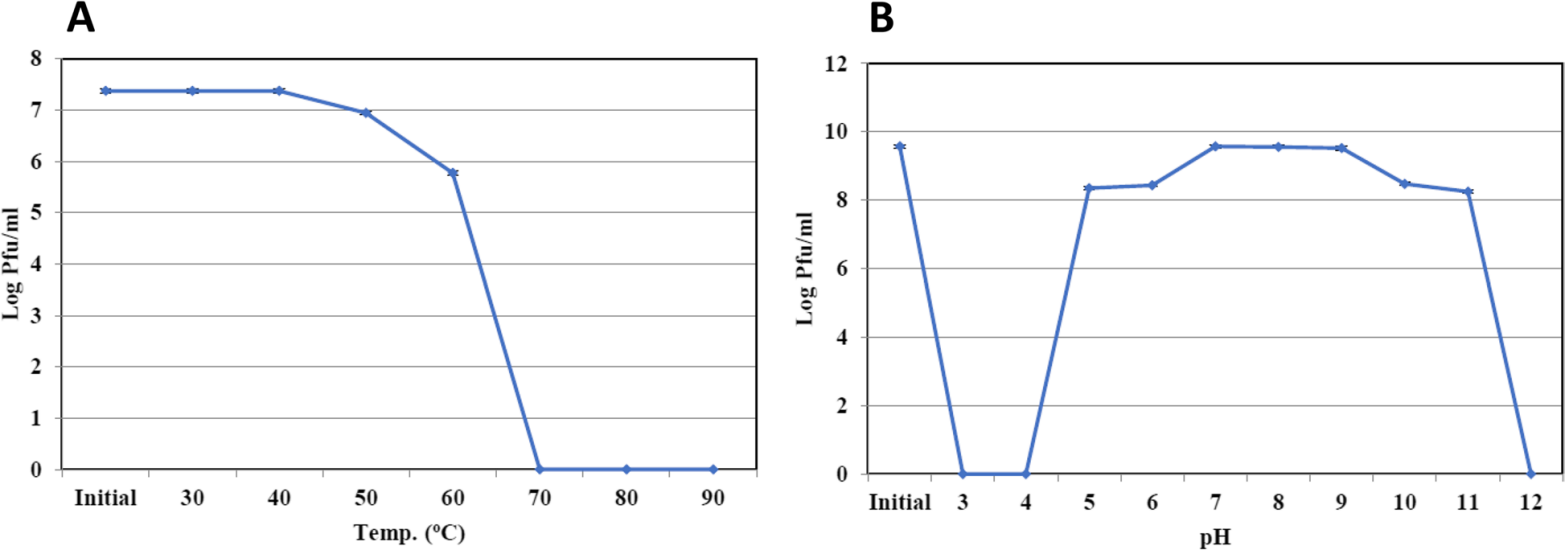
Effect of temperature and pH on the stability of *K. pneumoniae* KZag1 phage. **A** The stability of phage of *K. pneumoniae* Kzag1 at different temperatures. **B** The stability of KZag1 phage at different pH values. The number of phage was estimated by plaque assay using *K. pneumoniae*. Results are shown as means ± standard error

The stability of Zag1 phage was assessed under different temperature and pH conditions. The results indicated that the infectivity of KZag1phage remained largely unaffected by temperature, particularly up to 60 °C (Fig. 3A). The phage exhibited a survival rate ranging from 50 to 80% after exposure to temperatures of 70 °C for 10 min, suggesting its thermostability. However, at higher temperatures, KZag1 phage lost its infectivity and ability to lyse *K. pneumoniae* K9. Regarding pH stability, KZag1 phage demonstrated relatively stable behavior within the pH range of 6 to 8 (Fig. 3B). At pH levels of 11 or higher and pH levels of 4 or lower, Zag1 phage completely lost its infectivity. Notably, the phage exhibited greater stability and infectivity at pH 7. These findings highlight the resistance of KZag1 phage to temperature variations within a certain range and its sensitivity to extreme pH conditions. Understanding the stability of KZag1 phage under different environmental conditions is crucial for its potential applications and efficacy in controlling *K. pneumoniae* infections.

### In vitro assessment of KZag1 lytic activity against *K. pneumoniae* K9 biofilm

**Fig. 4:**
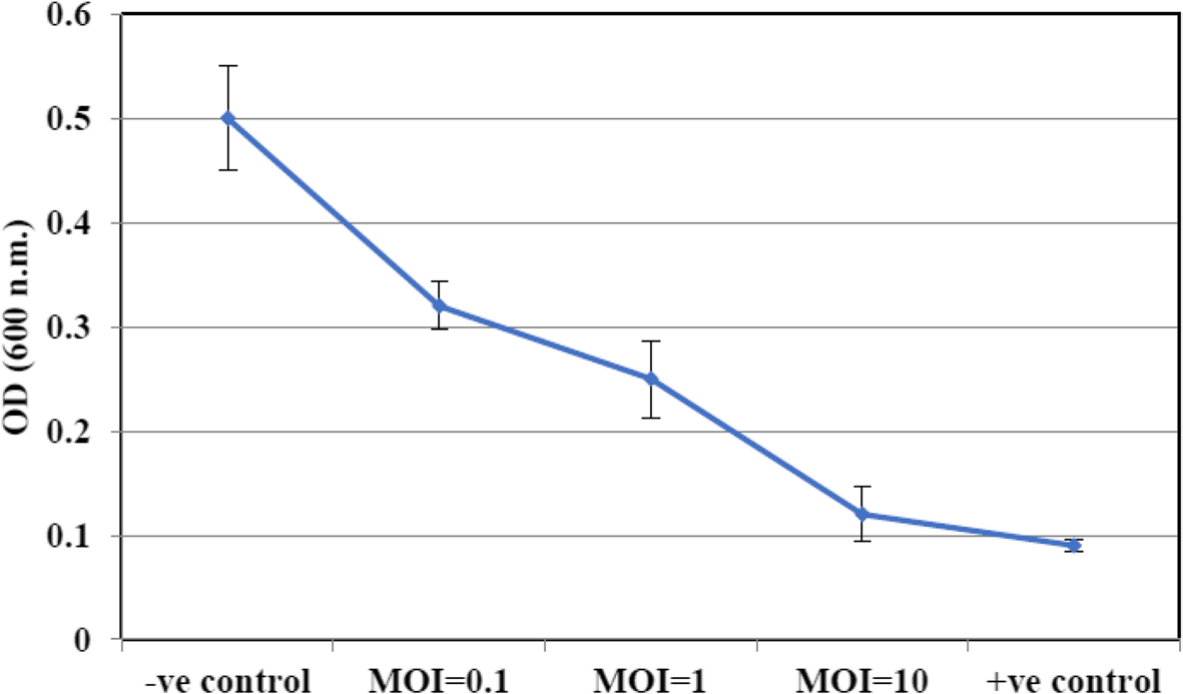
Phage Treatment of *K. pneumoniae* K9 Biofilm. The figure demonstrates the impact of Kzag1 phage treatment on *K. pneumoniae* bacterial biofilms using different multiplicities of infection (MOIs) of 0.1, 1 and10. Each data point on the graph represents the mean of three independent experiments. The results indicate the efficacy of phage treatment in reducing *K. pneumoniae* biofilm formation at varying MOI values

In vitro experiments were conducted to assess the lytic activity of KZag1 phage against a highly multidrug-resistant strain of *K. pneumoniae*. When *K. pneumoniae* K9 was used as the host in combination with the isolated Zag1 phage, remarkably different inhibition patterns were observed. After a 24-h incubation period, complete inhibition of bacterial growth was observed, demonstrating the potent inhibitory activity of KZag1 phage against *K. pneumoniae*. The activity of Zag1 phage against *K. pneumoniae* K9 biofilm formation was investigated using varying MOI values (0.1, 1, and 10). The results showed a significant decrease in the biofilm biomass when treated with Zag1 phage compared to the control group (Fig. 4) among the tested MOI values; an MOI of 10 exhibited the highest inhibition of *K. pneumoniae* K9biofilm formation by the phage (Fig. 4). These findings indicate the strong potential of Zag1 phage in targeting and reducing biofilm formation by *K. pneumoniae* K9. This suggests its potential as an effective biocontrol agent to combat *K. pneumoniae* infections. Further studies are warranted to explore its applicability and efficacy in real-world settings.

### Genomic features of KZag1 phage

**Fig. 5:**
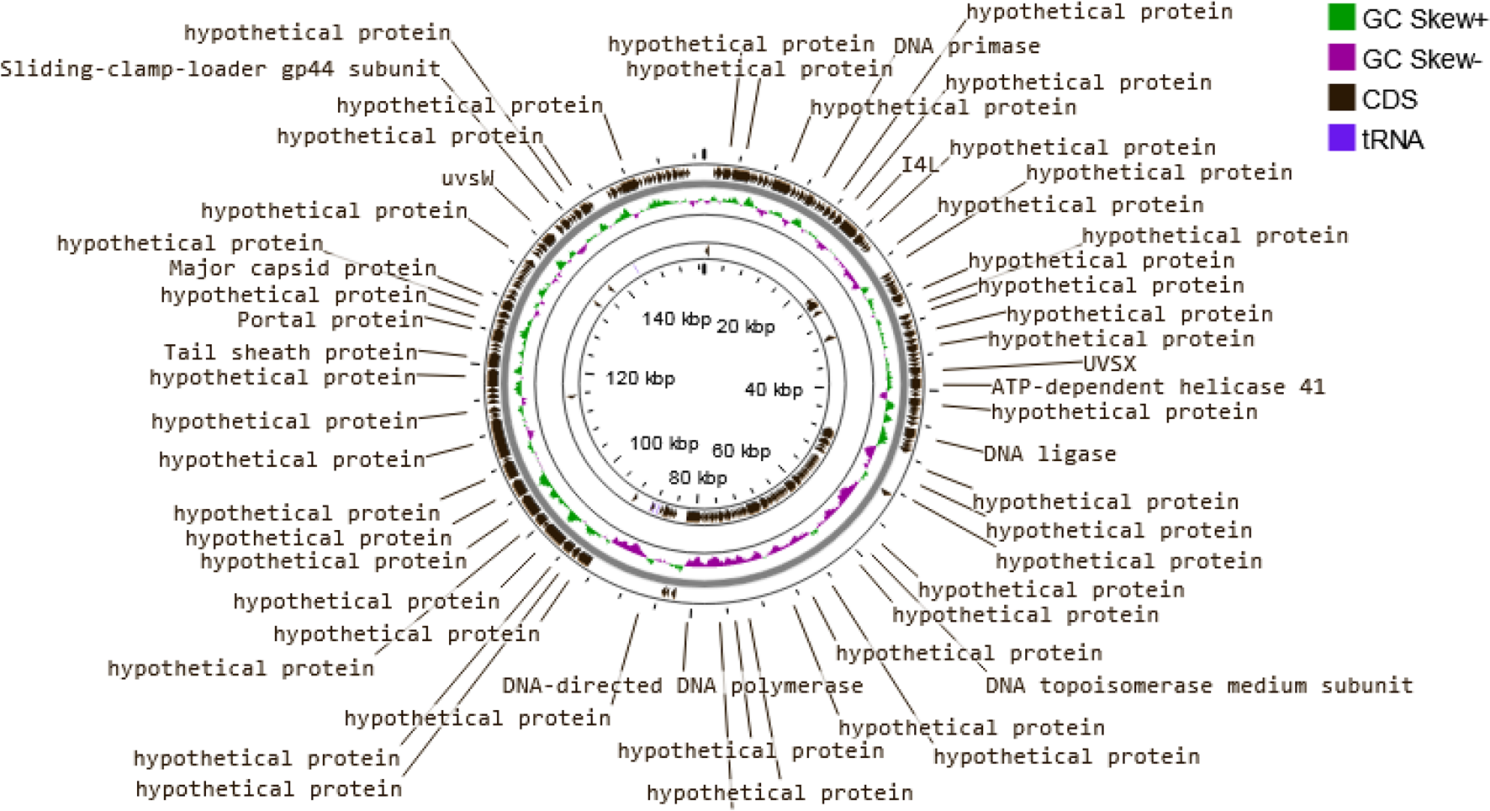
Whole genome map of phage Zag1. The figure displays the whole genome map of phage Zag1. The circular representation showcases the phage's genomic sequence, indicating the positions of various ORFs and functional elements. The map highlights key features and regions of interest within the phage's genetic structure

**Fig. 6:**
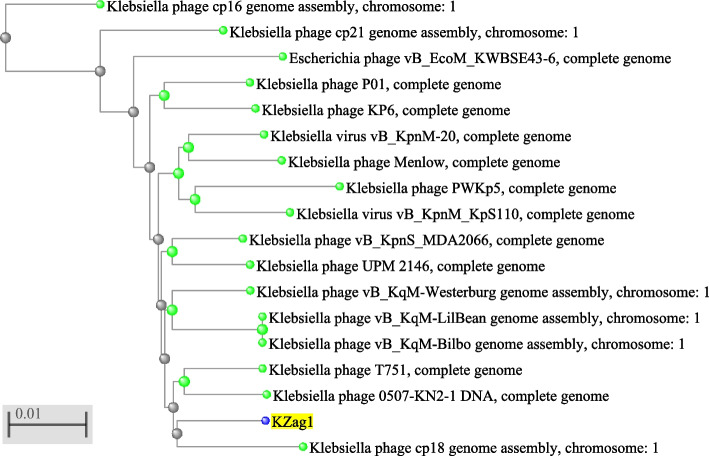
Phylogenetic tree depicting the evolutionary relationships among various bacteriophages, including *Klebsiella* phage cp16, cp21, P01, KP6, Menlow, PWKp5, vB_KpnM-20, vB_KpnM_KpS110, vB_KpnS_MDA2066, UPM 2146, vB_KqM-Westerburg, vB_KqM-LilBean, and vB_KqM-Bilbo, as well as *Escherichia* phage vB_EcoM_KWBSE43-6, *Klebsiella* phage T751, *Klebsiella* phage 0507-KN2-1 DNA, and KZag1. The scale bar represents a genetic distance corresponding to 0.01 substitutions per site, indicating minimal evolutionary divergence among the depicted phages

The genome of the KZag1 phage, belonging to the *Myoviridae* family, was analyzed in this study. The phage genome had a size of approximately 157 kilobase pairs (kbp) and was found to contain 202 predicted ORFs (Table 3). Comparative analysis revealed a high degree of similarity between the KZag1 phage and *Klebsiella* virus 0507KN21.The genomic analysis of KZag1 revealed several notable features (Fig. 5). The total length of the genome was determined to be approximately 157 kbp, which is relatively large compared to other characterized phages. Within this genome, 202 ORFs were identified and annotated. These ORFs encode proteins with various putative functions, including those involved in phage replication, DNA packaging, structural proteins, and host interaction. A comparative analysis of the KZag1 genome with *Klebsiella* virus 0507KN21 revealed significant similarity, as depicted in (Fig. 6). These two phages exhibited a high degree of nucleotide sequence identity and displayed conserved genomic organization, suggesting a close evolutionary relationship and shared ancestry. Phylogenetic analysis further elucidated this connection by revealing a close relationship between KZag1 and other phages: *Klebsiella* phage T751, and *Klebsiella* phage cp18 (Fig. 6). The scale bar of 0.01 indicates minimal evolutionary divergence among these phages, implying a recent common ancestor. This observation is reinforced by the fast minimum evolution method employed in the pairwise alignments, which underscores the genetic similarity and shared evolutionary history among these phages. The high number of ORFs in the KZag1 genome indicates a complex genetic composition. These ORFs likely play crucial roles in the phage's lifecycle, including host recognition, replication, and assembly. The presence of specific genes associated with DNA modification, recombination, and mobile genetic elements suggests the potential for genetic diversity and adaptation within the phage population. Furthermore, the similarity to *Klebsiella* virus 0507KN21 suggests a shared host range and similar strategies for infecting and propagating within *K. pneumoniae*. This finding is significant as it indicates that KZag1 may possess similar infectivity and therapeutic potential against *K. pneumoniae* biofilms. The genomic characterization of KZag1 provides valuable insights into its genetic makeup and potential functions. The presence of numerous ORFs, similarity to *Klebsiella* virus 0507KN21, and the identification of specific genes involved in phage-host interactions highlight the phage's ability to infect and replicate within *K. pneumoniae*. Further investigation into the specific functions and mechanisms of these genes will deepen our understanding of phage-host dynamics and facilitate the development of phage-based therapies against *K. pneumoniae* infections.

**Table 3:** Annotation table CDS Position of KZag1

ORF	CDS Position	BLAST Hit	E-Value
1	complement(120..887)	PHAGE_Klebsi_vB_KpnM_KpS110_NC_047932: ribonuclease; PP_00001; phage(gi100193)	0.0
2	929..1468	PHAGE_Klebsi_vB_KpnM_KpS110_NC_047932: hypothetical protein; PP_00002; phage(gi100194)	2.50e-129
3	1472..2242	PHAGE_Klebsi_vB_KpnM_KpS110_NC_047932: thymidylate synthase; PP_00003; phage(gi100195)	0.0
4	2229..3347	PHAGE_Serrat_vB_Sru_IME250_NC_042047: baseplate wedge; PP_00004; phage(gi100174)	0.0
5	3350..5677	PHAGE_Klebsi_vB_KpnM_KpS110_NC_047932: dihydrofolate reductase; PP_00005; phage(gi100197)	0.0
6	5680..5835	hypothetical; PP_00006	0.0
7	5832..6152	PHAGE_Klebsi_2146_NC_049472: hypothetical protein; PP_00007; phage(gi100002)	4.34e-73
8	6142..6423	PHAGE_Klebsi_2146_NC_049472: hypothetical protein; PP_00008; phage(gi100003)	1.03e-63
9	6521..7138	PHAGE_Klebsi_vB_KpnM_KpS110_NC_047932: hypothetical protein; PP_00009; phage(gi100201)	1.18e-152
10	7138..7776	PHAGE_Escher_vB_EcoM_KWBSE43_6_NC_048186: hypothetical protein; PP_00010; phage(gi100006)	1.28e-154
11	7885..8160	PHAGE_Klebsi_2146_NC_049472: hypothetical protein; PP_00011; phage(gi100006)	1.70e-61
12	8237..10615	PHAGE_Klebsi_vB_KpnM_KpS110_NC_047932: hypothetical protein; PP_00012; phage(gi100204)	0.0
13	10,669..11235	PHAGE_Klebsi_vB_KpnM_KpS110_NC_047932: hypothetical protein; PP_00013; phage(gi100205)	4.12e-135
14	11,299..11625	PHAGE_Klebsi_vB_KpnM_KpS110_NC_047932: ribonucleoside diphosphate reductase large subunit; PP_00014; phage(gi100207)	2.00e-75
15	11,701..12309	PHAGE_Escher_vB_EcoM_KWBSE43_6_NC_048186: hypothetical protein; PP_00015; phage(gi100014)	2.54e-152
16	12,320..12595	PHAGE_Klebsi_vB_KpnM_KpS110_NC_047932: hypothetical protein; PP_00016; phage(gi100003)	1.98e-61
17	12,592..13656	PHAGE_Klebsi_vB_KpnM_KpS110_NC_047932: hypothetical protein; PP_00017; phage(gi100004)	0.0
18	13,656..13868	PHAGE_Klebsi_vB_KpnM_KpS110_NC_047932: hypothetical protein; PP_00018; phage(gi100005)	1.50e-46
19	14,051..14539	PHAGE_Klebsi_vB_KpnM_KpS110_NC_047932: hypothetical protein; PP_00019; phage(gi100006)	4.28e-107
20	14,594..14782	PHAGE_Salmon_maane_NC_049508: hypothetical protein; PP_00020; phage(gi100091)	4.00e-39
21	14,779..15120	PHAGE_Pseudo_pf16_NC_041881: hypothetical protein; PP_00021; phage(gi100022)	1.36e-09
22	15,192..15986	PHAGE_Shigel_MK_13_NC_049455: hypothetical protein; PP_00022; phage(gi100191)	0.0
23	16,021..16728	PHAGE_Klebsi_vB_KpnM_KpS110_NC_047932: hypothetical protein; PP_00023; phage(gi100010)	4.52e-174
24	16,774..17616	PHAGE_Klebsi_vB_KpnM_KpS110_NC_047932: hypothetical protein; PP_00024; phage(gi100011)	0.0
25	17,704..19986	PHAGE_Klebsi_vB_KpnM_KpS110_NC_047932: hypothetical protein; PP_00025; phage(gi100012)	0.0
26	20,061..21164	PHAGE_Klebsi_vB_KpnM_KpS110_NC_047932: hypothetical protein; PP_00026; phage(gi100013)	0.0
27	21,174..21398	PHAGE_Klebsi_vB_KpnM_KpS110_NC_047932: hypothetical protein; PP_00027; phage(gi100014)	4.91e-48
28	21,493..21948	PHAGE_Escher_vB_EcoM_KWBSE43_6_NC_048186: hypothetical protein; PP_00028; phage(gi100028)	2.45e-109
29	21,955..22323	PHAGE_Klebsi_vB_KpnM_KpS110_NC_047932: hypothetical protein; PP_00029; phage(gi100016)	5.01e-87
30	complement(22,306..22686)	PHAGE_Klebsi_vB_KpnM_KpS110_NC_047932: hypothetical protein; PP_00030; phage(gi100017)	2.50e-89
31	complement(22,750..24360)	PHAGE_Klebsi_vB_KpnM_KpS110_NC_047932: hypothetical protein; PP_00031; phage(gi100018)	0.0
32	complement(24,861..25664)	PHAGE_Klebsi_2146_NC_049472: hypothetical protein; PP_00032; phage(gi100030)	0.0
33	25,714..26244	PHAGE_Klebsi_2146_NC_049472: hypothetical protein; PP_00033; phage(gi100031)	1.83e-129
34	26,216..26704	PHAGE_Klebsi_2146_NC_049472: u-spanin; PP_00034; phage(gi100032)	2.31e-116
35	26,743..27354	PHAGE_Klebsi_2146_NC_049472: endolysin; PP_00035; phage(gi100033)	2.45e-152
36	27,358..27486	hypothetical; PP_00036	0.0
37	27,630..27875	PHAGE_Klebsi_vB_KpnM_KpS110_NC_047932: hypothetical protein; PP_00037; phage(gi100024)	1.19e-54
38	27,868..28110	PHAGE_Klebsi_vB_KpnM_KpS110_NC_047932: hypothetical protein; PP_00038; phage(gi100025)	4.04e-51
39	28,120..28359	PHAGE_Klebsi_vB_KpnM_KpS110_NC_047932: hypothetical protein; PP_00039; phage(gi100026)	3.57e-53
40	28,456..29502	PHAGE_Klebsi_2146_NC_049472: hypothetical protein; PP_00040; phage(gi100038)	0.0
41	29,545..29949	PHAGE_Klebsi_2146_NC_049472: hypothetical protein; PP_00041; phage(gi100039)	6.10e-87
42	complement(29,973..30920)	PHAGE_Klebsi_vB_KpnM_KpS110_NC_047932: hypothetical protein; PP_00042; phage(gi100028)	0.0
43	30,974..31681	PHAGE_Klebsi_2146_NC_049472: DNA adenine methyltransferase; PP_00043; phage(gi100041)	1.77e-175
44	31,748..32482	PHAGE_Klebsi_vB_KpnM_KpS110_NC_047932: hypothetical protein; PP_00044; phage(gi100030)	2.21e-178
45	32,505..32816	PHAGE_Klebsi_vB_KpnM_KpS110_NC_047932: hypothetical protein; PP_00045; phage(gi100031)	1.26e-71
46	32,955..33866	PHAGE_Klebsi_2146_NC_049472: DNA helicase; PP_00046; phage(gi100044)	0.0
47	33,941..34603	PHAGE_Klebsi_2146_NC_049472: putative transcriptional regulator; PP_00047; phage(gi100045)	1.36e-164
48	34,603..35646	PHAGE_Klebsi_2146_NC_049472: HNH endonuclease; PP_00048; phage(gi100046)	0.0
49	35,643..36212	PHAGE_Escher_vB_EcoM_KWBSE43_6_NC_048186: recombination protein; PP_00049; phage(gi100049)	3.94e-139
50	36,209..36760	PHAGE_Escher_vB_EcoM_KWBSE43_6_NC_048186: putative single-stranded DNA binding protein; PP_00050; phage(gi100050)	1.53e-135
51	36,760..37278	PHAGE_Klebsi_2146_NC_049472: recombination protein; PP_00051; phage(gi100049)	1.22e-123
52	37,263..38348	PHAGE_Klebsi_vB_KpnM_KpS110_NC_047932: hypothetical protein; PP_00052; phage(gi100038)	0.0
53	38,326..38655	PHAGE_Klebsi_vB_KpnM_KpS110_NC_047932: hypothetical protein; PP_00053; phage(gi100039)	6.14e-75
54	38,662..40089	PHAGE_Klebsi_vB_KpnM_KpS110_NC_047932: hypothetical protein; PP_00054; phage(gi100040)	0.0
55	40,152..40487	PHAGE_Klebsi_vB_KpnM_KpS110_NC_047932: DNA adenine methyltransferase; PP_00055; phage(gi100041)	2.35e-79
56	40,501..40815	PHAGE_Klebsi_vB_KpnM_KpS110_NC_047932: hypothetical protein; PP_00056; phage(gi100042)	9.10e-71
57	40,934..42127	PHAGE_Klebsi_2146_NC_049472: minor tail protein; PP_00057; phage(gi100055)	0.0
58	42,124..42264	PHAGE_Klebsi_2146_NC_049472: minor tail protein; PP_00058; phage(gi100056)	1.21e-23
59	42,264..42476	PHAGE_Klebsi_vB_KpnM_KpS110_NC_047932: putative transcriptional regulator; PP_00059; phage(gi100045)	3.95e-46
60	42,478..42666	PHAGE_Klebsi_vB_KpnM_KpS110_NC_047932: HNH endonuclease; PP_00060; phage(gi100046)	1.74e-37
61	42,668..43009	PHAGE_Escher_vB_EcoM_KWBSE43_6_NC_048186: hypothetical protein; PP_00061; phage(gi100061)	2.78e-78
62	43,067..43675	PHAGE_Klebsi_2146_NC_049472: hypothetical protein; PP_00062; phage(gi100061)	2.98e-137
63	43,717..44274	PHAGE_Klebsi_2146_NC_049472: hypothetical protein; PP_00063; phage(gi100062)	1.10e-122
64	44,528..45949	PHAGE_Klebsi_2146_NC_049472: hypothetical protein; PP_00064; phage(gi100064)	0.0
65	45,946..46410	PHAGE_Entero_EspM4VN_NC_049384: hypothetical protein; PP_00065; phage(gi100018)	2.03e-33
66	46,410..46667	PHAGE_Klebsi_vB_KpnM_KpS110_NC_047932: exonuclease; PP_00066; phage(gi100048)	2.15e-54
67	46,633..46893	PHAGE_Klebsi_vB_KpnM_KpS110_NC_047932: recombination protein; PP_00067; phage(gi100049)	9.74e-58
68	46,883..47545	PHAGE_Klebsi_2146_NC_049472: capsid maturation protease; PP_00068; phage(gi100067)	6.11e-163
69	complement(47,548..49533)	PHAGE_Klebsi_2146_NC_049472: head morphogenesis protein; PP_00069; phage(gi100068)	0.0
70	complement(49,544..50932)	PHAGE_Klebsi_vB_KpnM_KpS110_NC_047932: tail fiber protein; PP_00070; phage(gi100052)	0.0
71	complement(50,929..51486)	PHAGE_Klebsi_vB_KpnM_KpS110_NC_047932: tail assembly protein; PP_00071; phage(gi100053)	1.27e-134
72	complement(51,501..52469)	PHAGE_Klebsi_2146_NC_049472: terminase small subunit; PP_00072; phage(gi100071)	0.0
73	52,523..53140	PHAGE_Klebsi_2146_NC_049472: hypothetical protein; PP_00073; phage(gi100072)	4.03e-151
74	complement(53,148..53345)	PHAGE_Klebsi_vB_KpnM_KpS110_NC_047932: tail length tape-measure protein; PP_00074; phage(gi100057)	1.82e-41
75	complement(53,348..53755)	PHAGE_Klebsi_2146_NC_049472: hypothetical protein; PP_00075; phage(gi100074)	4.21e-93
76	complement(53,766..54272)	PHAGE_Klebsi_vB_KpnM_KpS110_NC_047932: hypothetical protein; PP_00076; phage(gi100059)	8.08e-125
77	complement(54,265..54609)	PHAGE_Klebsi_vB_KpnM_KpS110_NC_047932: hypothetical protein; PP_00077; phage(gi100060)	3.93e-77
78	complement(54,672..55067)	PHAGE_Klebsi_vB_KpnM_KpS110_NC_047932: hypothetical protein; PP_00078; phage(gi100061)	3.06e-95
79	complement(55,064..55435)	PHAGE_Klebsi_vB_KpnM_KpS110_NC_047932: hypothetical protein; PP_00079; phage(gi100062)	3.67e-88
80	complement(55,500..55796)	PHAGE_Klebsi_2146_NC_049472: hypothetical protein; PP_00080; phage(gi100083)	7.52e-68
81	complement(55,796..56425)	PHAGE_Escher_vB_EcoM_KWBSE43_6_NC_048186: hypothetical protein; PP_00081; phage(gi100083)	3.01e-154
82	complement(56,418..57023)	PHAGE_Klebsi_2146_NC_049472: hypothetical protein; PP_00082; phage(gi100085)	1.74e-147
83	complement(57,020..57247)	PHAGE_Klebsi_2146_NC_049472: hypothetical protein; PP_00083; phage(gi100086)	1.74e-49
84	complement(57,247..57366)	PHAGE_Escher_vB_EcoM_KWBSE43_6_NC_048186: hypothetical protein; PP_00084; phage(gi100086)	5.79e-20
85	complement(57,410..57814)	PHAGE_Klebsi_2146_NC_049472: hypothetical protein; PP_00085; phage(gi100088)	7.03e-95
86	complement(57,818..58132)	PHAGE_Klebsi_vB_KpnM_KpS110_NC_047932: head morphogenesis protein; PP_00086; phage(gi100068)	4.24e-73
87	complement(58,132..58422)	PHAGE_Klebsi_vB_KpnM_KpS110_NC_047932: portal protein; PP_00087; phage(gi100069)	2.06e-63
88	complement(58,465..59796)	PHAGE_Klebsi_vB_KpnM_KpS110_NC_047932: terminase large subunit; PP_00088; phage(gi100070)	0.0
89	complement(59,798..61708)	PHAGE_Klebsi_vB_KpnM_KpS110_NC_047932: terminase small subunit; PP_00089; phage(gi100071)	0.0
90	complement(61,757..62383)	PHAGE_Klebsi_vB_KpnM_KpS110_NC_047932: hypothetical protein; PP_00090; phage(gi100072)	1.30e-156
91	complement(62,380..62865)	PHAGE_Klebsi_vB_KpnM_KpS110_NC_047932: hypothetical protein; PP_00091; phage(gi100073)	3.06e-117
92	complement(63,147..63650)	PHAGE_Escher_vB_EcoM_KWBSE43_6_NC_048186: hypothetical protein; PP_00092; phage(gi100095)	6.72e-107
93	complement(63,739..63960)	PHAGE_Klebsi_vB_KpnM_KpS110_NC_047932: hypothetical protein; PP_00093; phage(gi100075)	2.76e-45
94	complement(63,962..64771)	PHAGE_Klebsi_vB_KpnM_KpS110_NC_047932: hypothetical protein; PP_00094; phage(gi100076)	0.0
95	complement(64,750..64929)	PHAGE_Klebsi_vB_KpnM_KpS110_NC_047932: hypothetical protein; PP_00095; phage(gi100077)	4.21e-38
96	complement(65,174..65584)	PHAGE_Klebsi_vB_KpnM_KpS110_NC_047932: single-stranded-DNA-specific exonuclease; PP_00096; phage(gi100078)	1.59e-93
97	complement(65,562..65858)	PHAGE_Klebsi_2146_NC_049472: putative DNA polymerase; PP_00097; phage(gi100100)	3.54e-67
98	complement(65,911..67497)	PHAGE_Klebsi_2146_NC_049472: nicotinamide mononucleotide transporter; PP_00098; phage(gi100101)	0.0
99	complement(67,531..70287)	PHAGE_Klebsi_2146_NC_049472: hypothetical protein; PP_00099; phage(gi100102)	0.0
100	complement(70,390..70728)	PHAGE_Klebsi_vB_KpnM_KpS110_NC_047932: deoxynucleoside kinase; PP_00100; phage(gi100082)	1.23e-76
101	complement(70,739..70906)	PHAGE_Klebsi_Menlow_NC_047901: hypothetical protein; PP_00101; phage(gi100006)	5.62e-33
102	complement(70,903..71121)	PHAGE_Klebsi_Magnus_NC_049462: hypothetical protein; PP_00102; phage(gi100006)	4.93e-46
103	complement(71,121..71774)	PHAGE_Klebsi_Magnus_NC_049462: hypothetical protein; PP_00103; phage(gi100008)	2.16e-147
104	complement(71,771..72157)	PHAGE_Klebsi_Magnus_NC_049462: hypothetical protein; PP_00104; phage(gi100010)	4.07e-88
105	complement(72,223..72657)	PHAGE_Klebsi_Magnus_NC_049462: hypothetical protein; PP_00105; phage(gi100012)	6.27e-99
106	complement(72,662..73225)	PHAGE_Klebsi_vB_KpnM_KpS110_NC_047932: hypothetical protein; PP_00106; phage(gi100088)	1.17e-135
107	complement(73,242..74333)	PHAGE_Klebsi_Menlow_NC_047901: hypothetical protein; PP_00107; phage(gi100018)	0.0
108	complement(74,330..74449)	PHAGE_Klebsi_vB_KpnM_KpS110_NC_047932: putative HNH homing endonuclease; PP_00108; phage(gi100090)	2.35e-20
109	complement(74,644..75861)	PHAGE_Klebsi_Magnus_NC_049462: hypothetical protein; PP_00109; phage(gi100020)	0.0
110	complement(75,865..76245)	PHAGE_Klebsi_2146_NC_049472: hypothetical protein; PP_00110; phage(gi100113)	4.87e-89
111	complement(76,242..76445)	PHAGE_Klebsi_vB_KpnM_KpS110_NC_047932: hypothetical protein; PP_00111; phage(gi100093)	3.11e-42
112	complement(76,448..77332)	PHAGE_Klebsi_Menlow_NC_047901: hypothetical protein; PP_00112; phage(gi100028)	0.0
113	complement(77,332..77580)	PHAGE_Klebsi_Menlow_NC_047901: hypothetical protein; PP_00113; phage(gi100030)	1.56e-54
114	complement(77,642..78433)	PHAGE_Klebsi_Menlow_NC_047901: u-spanin; PP_00114; phage(gi100032)	0.0
115	complement(78,430..78780)	PHAGE_Klebsi_2146_NC_049472: hypothetical protein; PP_00115; phage(gi100118)	6.15e-81
116	complement(78,847..81843)	PHAGE_Klebsi_Magnus_NC_049462: holin; PP_00116; phage(gi100034)	0.0
117	81,943..82494	PHAGE_Klebsi_vB_KpnM_KpS110_NC_047932: hypothetical protein; PP_00117; phage(gi100099)	5.77e-130
118	82,841..83065	PHAGE_Klebsi_Menlow_NC_047901: hypothetical protein; PP_00118; phage(gi100042)	5.91e-50
119	83,134..83661	PHAGE_Klebsi_2146_NC_049472: DNA polymerase III alpha subunit; PP_00119; phage(gi100121)	1.13e-127
120	complement(83,743..84195)	PHAGE_Klebsi_Menlow_NC_047901: HNH endonuclease; PP_00120; phage(gi100046)	1.36e-101
121	complement(84,228..84686)	PHAGE_Klebsi_Menlow_NC_047901: exonuclease; PP_00121; phage(gi100048)	7.86e-109
122	complement(84,727..84879)	PHAGE_Klebsi_Menlow_NC_047901: putative single-stranded DNA binding protein; PP_00122; phage(gi100050)	2.86e-29
123	complement(84,876..86246)	PHAGE_Escher_vB_EcoM_KWBSE43_6_NC_048186: HNH homing endonuclease; PP_00123; phage(gi100129)	0.0
124	complement(86,316..86921)	PHAGE_Klebsi_vB_KpnM_KpS110_NC_047932: hypothetical protein; PP_00124; phage(gi100104)	6.33e-147
125	complement(87,247..87320)	tRNA	0.0
126	complement(87,443..87526)	tRNA	0.0
127	complement(88,112..88187)	tRNA	0.0
128	complement(88,198..88273)	tRNA	0.0
129	complement(88,281..88472)	PHAGE_Salmon_rabagast_NC_049499: hypothetical protein; PP_00125; phage(gi100191)	1.91e-38
130	complement(91,814..92383)	PHAGE_Klebsi_0507_KN2_1_NC_022343: hypothetical protein; PP_00126; phage(gi543171769)	3.59e-140
131	92,738..94519	PHAGE_Klebsi_0507_KN2_1_NC_022343: baseplate wedge subunit; PP_00127; phage(gi543171770)	0.0
132	94,503..95354	PHAGE_Klebsi_2146_NC_049472: putative baseplate hub protein; PP_00128; phage(gi100137)	0.0
133	95,359..96684	PHAGE_Klebsi_0507_KN2_1_NC_022343: hypothetical protein; PP_00129; phage(gi543171772)	0.0
134	96,736..99618	PHAGE_Klebsi_0507_KN2_1_NC_022343: putative tailspike protein; PP_00130; phage(gi543171773)	0.0
135	99,630..99902	PHAGE_Klebsi_vB_KpnM_KpS110_NC_047932: hypothetical protein; PP_00131; phage(gi100119)	2.80e-46
136	99,949..101295	PHAGE_Klebsi_0507_KN2_1_NC_022343: hypothetical protein RaK2_00525; PP_00132; phage(gi543171777)	9.31e-53
137	101,353..104010	PHAGE_Klebsi_0507_KN2_1_NC_022343: putative tail fiber protein; PP_00133; phage(gi543171774)	6.23e-106
138	104,080..106164	PHAGE_Klebsi_0507_KN2_1_NC_022343: tail spike protein head-binding protein; PP_00134; phage(gi543171775)	1.21e-29
139	106,379..108619	PHAGE_Klebsi_2146_NC_049472: major tail protein; PP_00135; phage(gi100145)	6.91e-33
140	108,630..108905	PHAGE_Erwini_PEp14_NC_016767: hypothetical protein; PP_00136; phage(gi374531865)	3.26e-10
141	109,001..113839	PHAGE_Klebsi_0507_KN2_1_NC_022343: vrlC protein; PP_00137; phage(gi543171778)	0.0
142	113,890..114138	PHAGE_Klebsi_vB_KpnM_KpS110_NC_047932: recombination endonuclease subunit D12; PP_00138; phage(gi100127)	1.97e-54
143	114,122..114460	PHAGE_Klebsi_0507_KN2_1_NC_022343: hypothetical protein; PP_00139; phage(gi543171779)	4.82e-74
144	114,447..115199	PHAGE_Klebsi_2146_NC_049472: prohead protease; PP_00140; phage(gi100150)	0.0
145	complement(115,228..115440)	PHAGE_Klebsi_Magnus_NC_049462: putative methyltransferase; PP_00141; phage(gi100107)	6.27e-45
146	115,502..116143	PHAGE_Klebsi_0507_KN2_1_NC_022343: neck protein; PP_00142; phage(gi543171781)	8.09e-156
147	116,146..116844	PHAGE_Klebsi_0507_KN2_1_NC_022343: proximal tail sheath stabilization; PP_00143; phage(gi543171782)	1.50e-175
148	116,847..117533	PHAGE_Klebsi_0507_KN2_1_NC_022343: terminase DNA packaging enzyme small subunit; PP_00144; phage(gi543171783)	5.73e-164
149	117,514..119730	PHAGE_Klebsi_0507_KN2_1_NC_022343: terminase subunit for DNA packaging, nuclease and ATPase; PP_00145; phage(gi543171784)	0.0
150	119,776..121671	PHAGE_Klebsi_0507_KN2_1_NC_022343: tail sheath protein; PP_00146; phage(gi543171785)	0.0
151	121,740..122195	PHAGE_Klebsi_0507_KN2_1_NC_022343: GIY-YIG endonuclease; PP_00147; phage(gi543171786)	8.88e-109
152	122,230..122763	PHAGE_Klebsi_0507_KN2_1_NC_022343: tail tube protein; PP_00148; phage(gi543171787)	9.75e-129
153	122,832..124514	PHAGE_Klebsi_0507_KN2_1_NC_022343: portal vertex protein of the head; PP_00149; phage(gi543171788)	0.0
154	124,560..124724	PHAGE_Klebsi_vB_KpnM_KpS110_NC_047932: hypothetical protein; PP_00150; phage(gi100140)	7.75e-31
155	124,734..125042	PHAGE_Klebsi_0507_KN2_1_NC_022343: putative prohead core protein; PP_00151; phage(gi543171789)	1.65e-65
156	125,053..125718	PHAGE_Klebsi_0507_KN2_1_NC_022343: putative prohead protease; PP_00152; phage(gi543171790)	6.09e-164
157	125,764..126618	PHAGE_Klebsi_0507_KN2_1_NC_022343: prohead core scaffold protein; PP_00153; phage(gi543171791)	0.0
158	126,713..128035	PHAGE_Klebsi_0507_KN2_1_NC_022343: phage major head protein/major capsid protein; PP_00154; phage(gi543171792)	0.0
159	128,119..128766	PHAGE_Salmon_SS9_NC_049458: hypothetical protein; PP_00155; phage(gi100178)	5.46e-162
160	128,826..129071	PHAGE_Klebsi_Menlow_NC_047901: pore-forming tail tip protein; PP_00156; phage(gi100139)	6.43e-54
161	129,068..129319	PHAGE_Klebsi_Magnus_NC_049462: pore-forming tail tip protein; PP_00157; phage(gi100139)	2.20e-39
162	129,742..129948	PHAGE_Klebsi_Menlow_NC_047901: major tail protein; PP_00158; phage(gi100145)	1.32e-41
163	129,929..130159	PHAGE_Klebsi_0507_KN2_1_NC_022343: hypothetical protein; PP_00159; phage(gi543171793)	4.17e-48
164	130,175..130612	PHAGE_Klebsi_2146_NC_049472: tail fibers protein; PP_00160; phage(gi100168)	1.83e-105
165	130,612..131052	PHAGE_Klebsi_0507_KN2_1_NC_022343: hypothetical protein; PP_00161; phage(gi543171795)	1.30e-102
166	131,054..131290	PHAGE_Klebsi_Menlow_NC_047901: portal protein; PP_00162; phage(gi100153)	6.57e-53
167	131,394..131693	PHAGE_Klebsi_2146_NC_049472: tail fibers protein; PP_00163; phage(gi100171)	1.47e-68
168	132,038..132481	PHAGE_Klebsi_0507_KN2_1_NC_022343: hypothetical protein; PP_00164; phage(gi543171796)	5.20e-105
169	132,505..132672	PHAGE_Klebsi_2146_NC_049472: baseplate wedge; PP_00165; phage(gi100174)	7.61e-33
170	132,707..133435	PHAGE_Klebsi_Magnus_NC_049462: hypothetical protein; PP_00166; phage(gi100163)	8.06e-176
171	complement(133,436..134092)	PHAGE_Klebsi_0507_KN2_1_NC_022343: hypothetical protein; PP_00167; phage(gi543171798)	5.48e-154
172	134,122..134622	PHAGE_Klebsi_vB_KpnM_KpS110_NC_047932: hypothetical protein; PP_00168; phage(gi100160)	4.67e-118
173	134,660..135124	PHAGE_Klebsi_0507_KN2_1_NC_022343: putative DNA repair/recombination protein UvsY; PP_00169; phage(gi543171800)	4.48e-109
174	135,124..135870	PHAGE_Klebsi_Magnus_NC_049462: tail fibers protein; PP_00170; phage(gi100171)	0.0
175	135,898..137415	PHAGE_Klebsi_vB_KpnM_KpS110_NC_047932: D5 protein; PP_00171; phage(gi100164)	0.0
176	complement(137,400..137783)	PHAGE_Klebsi_2146_NC_049472: hypothetical protein; PP_00172; phage(gi100181)	4.65e-89
177	138,107..138775	PHAGE_Klebsi_vB_KpnM_KpS110_NC_047932: hypothetical protein; PP_00173; phage(gi100166)	3.32e-164
178	138,861..139850	PHAGE_Klebsi_0507_KN2_1_NC_022343: sliding clamp holder; PP_00174; phage(gi543171805)	0.0
179	139,853..140275	PHAGE_Klebsi_0507_KN2_1_NC_022343: clamp holder for DNA polymerase; PP_00175; phage(gi543171806)	1.45e-100
180	140,304..140768	PHAGE_Klebsi_vB_KpnM_KpS110_NC_047932: hypothetical protein; PP_00176; phage(gi100169)	2.11e-112
181	140,785..141651	PHAGE_Klebsi_0507_KN2_1_NC_022343: hypothetical protein; PP_00177; phage(gi543171808)	0.0
182	141,723..142943	PHAGE_Klebsi_0507_KN2_1_NC_022343: hypothetical protein; PP_00178; phage(gi543171809)	0.0
183	143,281..145317	PHAGE_Klebsi_0507_KN2_1_NC_022343: hypothetical protein; PP_00179; phage(gi543171810)	0.0
184	145,354..145728	PHAGE_Klebsi_0507_KN2_1_NC_022343: hypothetical protein; PP_00180; phage(gi543171811)	2.23e-78
185	145,794..146546	PHAGE_Klebsi_0507_KN2_1_NC_022343: hypothetical protein; PP_00181; phage(gi543171815)	7.76e-177
186	146,611..146967	PHAGE_Klebsi_0507_KN2_1_NC_022343: hypothetical protein; PP_00182; phage(gi543171816)	3.73e-78
187	146,975..147118	PHAGE_Klebsi_Magnus_NC_049462: hypothetical protein; PP_00183; phage(gi100205)	1.62e-25
188	147,111..149309	PHAGE_Klebsi_0507_KN2_1_NC_022343: hypothetical protein; PP_00184; phage(gi543171817)	0.0
189	149,354..149677	PHAGE_Klebsi_0507_KN2_1_NC_022343: putative acyl carrier protein; PP_00185; phage(gi543171818)	3.82e-70
190	149,992..150228	PHAGE_Klebsi_vB_KpnM_KpS110_NC_047932: hypothetical protein; PP_00186; phage(gi100180)	2.24e-48
191	150,283..150651	PHAGE_Klebsi_0507_KN2_1_NC_022343: hypothetical protein; PP_00187; phage(gi543171819)	2.91e-85
192	150,671..150850	PHAGE_Klebsi_vB_KpnM_KpS110_NC_047932: ATP-dependent Clp protease; PP_00188; phage(gi100182)	2.20e-34
193	150,975..151424	PHAGE_Klebsi_0507_KN2_1_NC_022343: phage associated protein; PP_00189; phage(gi543171821)	2.45e-107
194	151,503..152189	PHAGE_Klebsi_0507_KN2_1_NC_022343: hypothetical protein; PP_00190; phage(gi543171822)	2.05e-168
195	152,189..152533	PHAGE_Klebsi_0507_KN2_1_NC_022343: hypothetical protein; PP_00191; phage(gi543171823)	4.93e-80
196	152,583..153323	PHAGE_Klebsi_0507_KN2_1_NC_022343: hypothetical protein; PP_00192; phage(gi543171824)	0.0
197	153,372..154079	PHAGE_Klebsi_0507_KN2_1_NC_022343: ImpD; PP_00193; phage(gi543171825)	2.96e-177
198	154,076..154351	PHAGE_Klebsi_Menlow_NC_047901: hypothetical protein; PP_00194; phage(gi100233)	1.15e-60
199	154,332..154658	PHAGE_Serrat_vB_Sru_IME250_NC_042047: tail fibers protein; PP_00195; phage(gi100168)	1.18e-61
200	154,655..154969	PHAGE_Klebsi_Menlow_NC_047901: hypothetical protein; PP_00196; phage(gi100237)	1.06e-71
201	complement(154,997..155113)	PHAGE_Klebsi_0507_KN2_1_NC_022343: hypothetical protein; PP_00198; phage(gi543171827)	3.12e-20
202	155,087..155365	PHAGE_Klebsi_Menlow_NC_047901: NAD dependent DNA ligase subunit A; PP_00197; phage(gi100239)	2.02e-59

In the table legend, The "ORF" column denotes the Open Reading Frame (ORF) number, serving as a unique identifier for each ORF. The "CDS Position" column indicates the position of the coding sequence (CDS) within the genome. In the "BLAST Hit" column, annotations or descriptions of sequence similarities identified through BLAST are provided, often specifying the source organism and the function or predicted function of the sequence. The "E-Value" column presents the Expect value (E-value), a parameter quantifying the statistical significance of sequence similarity. A lower E-value indicates a more significant match, with the number of expected chance hits decreasing accordingly

## Discussion

The present study focused on the analysis of a novel bacteriophage, KZag1, which specifically infects biofilms formed by *K. pneumoniae*. The research aimed to characterize the genome sequence and investigate the properties and potential applications of this phage. The findings contribute to our understanding of phage-host interactions and provide valuable insights for the development of phage-based therapies against *K. pneumoniae* biofilms. The first important aspect examined in this study was the sensitivity of *K. pneumoniae* strains to different antibiotics. The results revealed a high level of resistance to multiple antibiotics, indicating the presence of MDR strains. This observation aligns with previous reports highlighting the challenge of treating *K. pneumoniae* infections due to antibiotic resistance. The emergence of MDR strains poses a significant threat to public health, underscoring the urgent need for alternative treatment strategies [36]. To address this issue, the researchers isolated and characterized KZag1, a bacteriophage specifically targeting *K. pneumoniae* biofilms. The phage was successfully isolated from an enrichment culture containing *K. pneumoniae*, and its lytic activity against the bacterium was confirmed through spot and plaque assays. The morphological characterization using transmission electron microscopy revealed that KZag1 belongs to the *Myoviridae* family, possessing icosahedral heads and contractile tails. This classification provides important insights into the phage's structural characteristics, which may influence its infectivity and interaction with the host bacterium [37]**.** The host range analysis demonstrated that KZag1 exhibited strong lytic activity against *K. pneumoniae* strains but did not infect other tested bacterial strains, indicating a narrow host range. The specificity of bacteriophages towards certain bacterial hosts is a well-documented phenomenon and is influenced by various factors such as surface receptors, bacterial cell wall structures, and immune evasion mechanisms. In the case of bacteriophage KZag1, its strong lytic activity against *K. pneumoniae* strains while not infecting other tested bacterial strains suggests a high degree of specificity towards *K. pneumoniae*. This specificity can be attributed to the presence of specific receptor sites on the surface of *K. pneumoniae* cells that are recognized by the phage's tail fibers or other structural proteins. The absence or structural differences of these receptor sites in other bacterial species may prevent the attachment and subsequent infection by the bacteriophage. Furthermore, the genomic makeup of bacteriophage KZag1 likely contains genes encoding proteins that specifically target and interact with components unique to *K. pneumoniae*, contributing to its strong lytic activity against this bacterial species. While the exact mechanisms underlying the narrow host range of bacteriophage KZag1 may require further investigation, previous studies on phage-host interactions have demonstrated similar patterns of specificity, emphasizing the importance of understanding the molecular determinants driving bacteriophage infectivity. While a broad host range is desirable for therapeutic phages, narrow host specificity can still be advantageous in certain scenarios [38]. By targeting *K. pneumoniae* specifically, KZag1 may offer a more precise and effective approach for treating *K. pneumoniae* biofilm-related infections without disrupting the beneficial microbial flora. The one-step growth curve analysis provided insights into the dynamics of phage infection. The latent period, generation time, and burst size of KZag1 were determined. The relatively short latent period of 20 min followed by a rise period of 35 min suggests that the phage has a rapid replication cycle. The burst size, which ranged from 83 to 100 phages per cell, indicates the potential for efficient phage-mediated lysis of *K. pneumoniae* biofilms. These findings provide valuable information for optimizing the therapeutic application of KZag1, such as determining the appropriate timing and dosage for effective treatment. Phage stability is a crucial factor to consider when developing phage-based therapies [39]**.** The study investigated the effect of temperature and pH on the stability of KZag1. The results demonstrated that the phage remained infective and capable of lysing *K. pneumoniae* even after exposure to temperatures up to 60 °C, indicating its thermostability. Additionally, KZag1 exhibited optimal stability within a pH range of 6–8. However, extreme pH conditions, either highly acidic or alkaline, resulted in the loss of phage infectivity. These findings highlight the importance of considering environmental conditions when utilizing KZag1 as a therapeutic agent [39]. The characterization of KZag1 and its demonstrated activity against *K. pneumoniae* K9 biofilms contribute to the growing body of knowledge on phage-based therapies. By specifically targeting biofilms, which are notoriously resistant to conventional antibiotics, KZag1 offers a potential alternative for combating *K. pneumoniae* infections. The narrow host range of KZag1 may limit its application to *K. pneumoniae* strains only, but it also reduces the risk of impacting the natural microbiota. Moreover, the phage's thermostability and stability within a physiological pH range enhance its potential for practical application. The analysis of the KZag1 phage genome, with a size of approximately 157 kilobase pairs (kbp) and belonging to the *Myoviridae* family, revealed several intriguing findings. The presence of 202 predicted ORFs within the genome suggests a complex genetic composition with various functional elements. The results of our phylogenetic analysis shed light on the evolutionary relationships between KZag1 and *Klebsiella* virus 0507KN21, along with other related *Klebsiella* phages: *Klebsiella* phage T751, and *Klebsiella* phage cp18, suggesting a close evolutionary connection and shared genetic ancestry [40]. Such similarity in genomic organization and sequence conservation suggests common strategies for infecting and interacting with *K. pneumoniae*, the host bacterium. These findings offer valuable insights into the evolutionary dynamics of bacteriophages within the *Klebsiella* genus, crucial for understanding their evolutionary trajectories, host specificity, and potential applications in phage therapy and biotechnology. The abundance of ORFs in the KZag1 genome provides insight into the genetic diversity and complexity of the phage. These ORFs likely encode proteins involved in essential processes such as phage replication, assembly, DNA packaging, and host interaction. Detailed functional analysis of these ORFs can shed light on the mechanisms underlying the phage's lifecycle and its interaction with the host bacterium. Furthermore, the identification of specific genes associated with DNA modification, recombination, and mobile genetic elements within the KZag1 genome suggests potential mechanisms for genetic variation and adaptation. These elements may contribute to the phage's ability to evolve and overcome bacterial defense mechanisms, as well as facilitate the exchange of genetic material with other phages or bacterial hosts. The similarity to other *Klebsiella* phages, including *Klebsiella* virus 0507KN21, is particularly significant in the context of phage-based therapies [41]. The close genetic relatedness between these phages suggests that they may share similar host ranges and infection mechanisms. This similarity provides promising prospects for the development of phage cocktails or combination therapies targeting *K. pneumoniae* infections, including those associated with biofilms. Further studies are warranted to explore the functional significance of the identified ORFs and their role in phage-host interactions. Comparative genomics and proteomics approaches can provide insights into the shared and unique features of the related *Klebsiella* phages [42, 43]**.** Additionally, assessing the efficacy of these phages in biofilm eradication and infection control is crucial to evaluate their potential as therapeutic agents. In conclusion this study provides valuable insights into the characterization and potential therapeutic application of KZag1, a novel bacteriophage targeting biofilms formed by *K. pneumoniae*. The findings contribute to our understanding of phage-host interactions and highlight the potential of phage-based therapies as an alternative to combat antibiotic-resistant infections. Further research and clinical trials are warranted to evaluate the efficacy and safety of KZag1 and its potential integration into clinical practice for the treatment of *K. pneumoniae* biofilm-related infections.

## Data Availability

Sequence of the analyzed strain has been deposited in the GenBank nucleotide sequence database at the National Library of Medicine, National Center for Biotechnology Information (NCBI). The assigned accession number for the sequence is OP942216, and it can be accessed at [The GenBank accession number link provided: https://www.ncbi.nlm.nih.gov/nucleotide/OP942216.1].
